# Supplementary material for: PLOS Computational Biology 2016 Reviewer and Editorial Board Thank You
Source: PLoS Comput Biol. 2017 Mar 20;13(3):e1005442. doi: 10.1371/journal.pcbi.1005442 (PMC5358731; doi:10.1371/journal.pcbi.1005442)
Supplement: S1 Editor List — (PDF) [file pcbi.1005442.s001.pdf]

PLOS | COMPUTATIONAL BIOLOGY

PLOS Computational Biology would like to thank all those who served on the Editorial Board in 2016:

Mark Alber
Samuel Alizon
Stefano Allesina
Russ Altman
Rustom Antia
Becca Asquith
Anand Asthagiri
Joseph Ayers
Patricia Babbitt
Joel Bader
Daniel Beard
Jeff Beck
Niko Beerenwinkel
Nir Ben-Tal
Carl Bergstorm
Matthias Bethge
Kim Blackwell
Gunnar Blohm
Sebastian Bonhoeffer
Elhanan Borenstein
Peer Bork
Philip Bourne
Rachel Brem
Steven Brenner
Kevin Bretonnel Cohen
James Briggs
Daniel Bush
Kevin Chen
Shi-Jie Chen
Jean Daunizeau
Rob De Boer
Bert de Groot
Gustavo Deco
Scott Diamond
Jörn Diedrichsen
Roland Dunbrack Jr.
Wolfgang Einhäuser
Jonathan Eisen
Michael Eisen
David Eisenberg
Arne Elofsson
Bard Ermentrout
Aldo Faisal
Neil Ferguson
Matthew Ferrari
Jacquelyn Fetrow
Ernest Fraenkel
Christophe Fraser
James Gallo
Paul Gardner
Robert Gentleman
Lyle Graham
Helmut Grubmüller
Roderic Guigo
Boris Gutkin
Adrian Haith
Jason Haugh
Claus Hilgetag
Lilia Iakoucheva
Ilya Ioshikhes
Lars Jensen
Maricel Kann
Rachel Karchin
Alon Keinan
Ozlem Keskin
Roy Kishony
Katia Koelle
Natalia Komarova
Konrad Kording
Sergei Kosakovsky Pond
Nikolaus Kriegeskorte
Peter Latham
Douglas Lauffenburger
Thomas Lengauer
Christina Leslie
Justin Lessler
Michael Levitt
Fran Lewitter
Jennifer Listgarten
Dennis Livesay
James Lloyd-Smith
Feilim Mac Gabhann
Jakob Macke
Alexander MacKerell
Philip Maini
Costas Maranas
Daniele Marinazzo
Scott Markel
Florian Markowetz
Marc Marti-Renom

Frederick Matsen IV
John Mattick
Andrew McCulloch
Alice McHardy
Martin Meier-Schellersheim
Daniel Mietchen
Satoru Miyano
Alexandre Morozov
Quaid Morris
Abigail Morrison
Qing Nie
Jens Nielsen
William Noble
Ruth Nussinov
Yanay Ofran
Uwe Ohler
Jill O'Reilly
Christine Orengo
Francis Ouellette
Christos Ouzounis
Jason Papin
Mercedes Pascual
Bjoern Peters
Timothée Poisot
Andreas Prlic
Teresa Przytycka
Marco Punta
Predrag Radivojac
Christopher Rao
Benjamin Raphael
Jennifer Reed
Roland Regoes
Isidore Rigoutsos
Burkhard Rost
Andrey Rzhetsky
Marcel Salathé
Andrej Sali
Jeffrey Saucerman
Avner Schlessinger
Dina Schneidman
David Searls
Mona Singh
Donna Slonim
Olaf Sporns
Jorg Stelling
Emad Tajkhorshid
Kai Tan
Mark Tanaka
Amos Tanay
Frédéric Theunissen
Denis Thieffry
Olga Troyanskaya
Greg Tucker-Kellogg
David van der Spoel
Erik van Nimwegen
Christian von Mering
Rebecca Wade
Edwin Wang
Guanghong Wei
Claus Wilke
Lingchong You
Weixiong Zhang
Sheng Zhong
Xianghong Jasmine Zhou
