# Supplementary material for: PLOS Computational Biology 2016 Reviewer and Editorial Board Thank You
Source: PLoS Comput Biol. 2017 Mar 20;13(3):e1005442. doi: 10.1371/journal.pcbi.1005442 (PMC5358731; doi:10.1371/journal.pcbi.1005442)
Supplement: S1 Guest Editor List — (PDF) [file pcbi.1005442.s002.pdf]

PLOS | COMPUTATIONAL BIOLOGY

PLOS Computational Biology would like to thank all those who served as a Guest Associate Editor in 2016:

Christoph Adami
Reka Albert
Theodore Alexandrov
Samuel Alizon
Stefano Allesina
Patrick Aloy
Grégoire Altan-Bonnet
Jörn Anemüller
Lars Arvestad
Oleg Aslanidi
Gabor Balazsi
David Balding
Petr Baldrian
Ziv Bar-Joseph
Patrick Barth
David Basanta
Danielle Bassett
Saonli Basu
Francesco Battaglia
Catherine Beauchemin
Ulrik Beierholm
Matthew Bennett
Aviv Bergman
Max Berniker
Silvia Blemker
Norbert Boeddeker
Richard Bonneau
Anne-Laure Boulesteix
Kristin Branson
Yana Bromberg
Nicolas Brunel
Javier Buceta
Kelly Burrowes
Daniel Bush
Daniel Butts
Patrick Cahan
Claudine Chaouiya
Steven Chase
Gary Churchill
Radoslaw Cichy
Otto X. Cordero
Iain Couzin
Attila Csikász-Nagy
Thomas Dandekar
Paul de Bakker
Charlotte Deane
Diego Di Bernardo
Jochen Ditterich
Olaf Doessel
Nikolay Dokholyan
Dirk Drasdo
Patrick Drew
Eva Dyer
Leah Edelstein-Keshet
Matthew Ferrari
Sarel Fleishman
Andre Franke
Feng Fu
Sebastian Funk
Randy Gallistel
James Gallo
Sergey Gavrilets
Karl Gegenfurtner
Samuel Gershman
Dirk Gillespie
Tucker Gilman
Anthony Gitter
Christophe Godin
Richard Goldstein
Dmitry Gordenin
Jeff Gore
Raphael Gottardo
Julian Gough
Frederik Graw
Elizabeth Grice
Attila Gursoy
Ralf Haefner
Adrian Haith
Turkan Haliloglu
Betz Halloran
Andreas Handel
Matthew Hartfield
Moritz Helmstaedter
Charlotte Hemelrijk
Thomas Höfer
Hans Hofmann
Thomas House
Haiyan Huang
Xuhui Huang
Alex Huk

Oleg Igoshin
Donald Jacobs
Robert Jacobs
Mohsin Jafri
Saad Jbabdi
Robert Jernigan
Eric Jonas
Suckjoon Jun
Andrey Kajava
Christoph Kaleta
Lukas Käll
Ingmar Kanitscheider
Peter Kasson
Michael Keiser
Melissa Kemp
Philip Kim
Isaac Klapper
Stefan Klumpp
Jacob Koella
Roger Kouyos
Gabriel Kreiman
Nikolaus Kriegeskorte
David Ku
Liana Lareau
Boris Lenhard
Andre Levchenko
Simon Levin
Herbert Levine
Yaakov Levy
Nathan Lewis
Rune Linding
Andreas Linninger
Jin Liu
James Lloyd-Smith
James Locke
Radhakrishnan Mahadevan
Pascal Mamassian
Yael Mandel-Gutfreund
Daniele Marinazzo
Debora Marks
Alison Marsden
Sergei Maslov
Thomas Matthews
Laoise McNamara
Ron Meir
Roeland Merks
Richard Michod
Gabriel Mindlin
Mohammad Mofrad
James Moore
Burkhard Morgenstern
Andrew Mugler
Viktor Müller
Chad Myers
Shikha Nangia
Israel Nelken
Gregor Neuert
Qing Nie
Michael Nikolaou
Michael Nilges
Jill O'Reilly
Jorge Pacheco
Adam Packer
Bernhard Palsson
Alexander Panfilov
Smita Patel
Arvind Pathak
Kiran Patil
Shayn Peirce
David Penny
Alan Perelson
Darcy Peterka
Thomas Pfeiffer
Jonathan Pillow
Igor Pivkin
Joshua Plotkin
Ashok Prasad
Stephan Preibisch
Nathan Price
Przemyslaw Prusinkiewicz
Peng Qiu
Magnus Rattray
Roland Regoes
Alan Rein
Cynthia Reinhart-King
Olivier Restif
Angela Reynolds
Thomas Rich
Gerard Ridgway
Arnd Roth
Robert Russell
Julio Saez-Rodriguez
Maneesh Sahani
Howard Salis
Francisco Santos
Herbert Sauro
Jeffrey Schank
Tamar Schlick
Santiago Schnell
Paul Schrater
Gideon Schreiber
Joost Schymkowitz

Timothy Secomb
Ryan Senger
Oliver Serang
Anil Seth
Reza Shadmehr
Eugene Shakhnovich
Brian Shoichet
Stanislav Shvartsman
Markus Siegel
Saurabh Sinha
David Smith
Maurice Smith
Yun Song
Rosangela Sozzani
Johannes Spelbrink
David Sprinzak
Michael Stadler
Matthew Stephens
Volker Steuber
Ian Stevenson
Ann Stock
Pavel Sumazin
De Witt Sumners
Fabian Theis
Frédéric Theunissen
Michael Tildesley
Marcus Tindall
Gašper Tkačik
Sina Tootoonian
Arne Traulsen
Jochen Triesch
Ten Tusscher
David Umulis
Francisco Valero-Cuevas
Mark van Rossum
Koen Vervaeke
Martin Vingron
Thomas Voets
Eberhard Voit
Niels Volkmann
Anders Wallqvist
Ting Wang
Kunlin Wei
Leor Weinberger
Franz Weissing
Lonnie Welch
Benjamin Werner
Daniel Wilson
Dominik Wodarz
Joao Xavier
Yu Xia
Lei Xie
Esti Yeger-Lotem
Byron Yu
Henggui Zhang
Zhongming Zhao
Yishao Zhou
