# Supplementary material for: PLOS Computational Biology 2016 Reviewer and Editorial Board Thank You
Source: PLoS Comput Biol. 2017 Mar 20;13(3):e1005442. doi: 10.1371/journal.pcbi.1005442 (PMC5358731; doi:10.1371/journal.pcbi.1005442)
Supplement: S1 Reviewer List — (PDF) [file pcbi.1005442.s003.pdf]

PLOS Computational Biology would like to thank all those who reviewed on behalf of the journal in 2016:

Daniel Aalberts
Daniel Abankwa
Henry Abarbanel
Kotb Abdelmohsen
Amir Abdollahi
Pia Abel Zur Wiesch
Hermann Aberle
Romesh Abeysuriya
Murat Acar
Luigi Acerbi
Daniel Acuna
John Adam
Christoph Adami
Wendy Adams
Sergiy Afonin
Nasrin Afzal
Pratul Agarwal
Loranne Agius
Antoni Aguilar-Mogas
Zulfiqar Ahmad
Alaa Ahmed
Hasan Ahmed
Natalie Ahn
Sebastian Ahnert
Stuart Aitken
Marco Ajelli
Thomas Akam
Ilya Akberdin
Eyal Akiva
Bülent Aksoy
John Albeck
Reka Albert
Istvan Albert
Leonidas Alexopoulos
Emil Alexov
Babak Alipanahi
Ash Alizadeh
Mohammad Alkhamis
Jun Allard
Carsten Allefeld
Genevera Allen
Eivind Almaas
Daniel Almonacid
Uri Alon
David Alonso
Petko Alov
Patrick Aloy
Katherine Alser
Grégoire Altan-Bonnet
William Altemeier
Adrian Altenhoff
Christian Althaus
Benjamin Althouse
Russ Altman
Philipp Altrock
Vikram Alva
Veronica Alvarez
Francisco Alvarez-Leefmans
Rommie Amaro
Ettore Ambrosini
Sophia Ananiadou
Bharath Ananthasubramaniam
Alexander Anderson
Jean-Baptiste André
Simon Andrews
Steven Andrews
Ioannis Androulakis
Danny Antaki
Zador Anthony
Maciek Antoniewicz
Marco Antoniotti
Christopher Antos
Haroon Anwar
Stefano Anzellotti
Shinya Aoi
Mikio Aoi
Kevin Aquino
Arturo Araujo
Marcos Araúzo-Bravo
Leonardo Arbiza
Murat Arcak
Julia Arciero
Yair Argon
Gil Ariel
Nimalan Arinaminpathy
Clement Arnarez
David Arnosti
Javier Arsuaga
Ronan Arthur
Kenta Asahina

Peter Ashcroft
Go Ashida
Hiroshi Ashikaga
Peter Ashwin
Michael Assaf
Florencia Assaneo
Collins Assisi
Katherine Atkins
Charles Auffray
Alexander Aulehla
Erin Austin
Joseph M. Autry
Bruno Averbeck
Ferhat Ay
Ramy Aziz
Marc Baaden
Nils Baas
M. Madan Babu
Mohan Babu
Marco Bacci
Stephen Baccus
Nadine Bachmann
Gary Bader
Jonathan Badger
Neda Bagheri
Justin Bahl
Bahador Bahrami
Lu Bai
Wyeth Bair
Chris Bakal
Michelle Baker
Gabor Balazsi
Eva Balsa-Canto
Sourav Bandyopadhyay
Sourav Banerjee
Rahul Banerjee
Julio Banga
Edward Banigan
Brittany Bannish
Shweta Bansal
Gyorgy Barabas
Omri Barak
Pavel Baranov
Yoseph Barash
Chris Barclay
Izhar Bar-Gad
Allison Barner
Jose Barral
Perdita Barran
Alain Barrat
Ernest Barreto
Adam Barrett
Jeffrey Barrick
Andrew Barron
Patrick Barth
Istvan Bartha
John Barton
Anastasia Baryshnikova
Patricia Bassereau
Alex Bateman
Brice Bathelier
Kayhan Batmanghelich
Brendan Battersby
Tuncay Baubec
Chris Bauch
Anaïs Baudot
Carl Bauer
Jan Baumbach
Maxim Bazhenov
Jason Bazil
Andrew Beam
Mark Beaumont
Oliver Beckstein
Michael Beer
John Beggs
Marcelo Behar
Afshin Beheshti
Tim Beißbarth
Robert Belshaw
Joost Beltman
Pedro Beltrao
Martine Ben Amar
Jan Benda
Sean Bendall
Andreas Bender
Shifra Ben-Dor
Klaus Benndorf
Matthew Bennett
Loisa Bennetto
Richard Benninger
Panayiotis Benos
Andrea Benucci
Lubica Benuskova
Ludek Berec
Omer Berenfeld
Philipp Berens
Igor Berezovsky
Aviv Bergman
Christina Bergonzo
Antony Beris
Spring Berman
Alberto Bernacchia

Denzil Bernard
Max Berniker
Julien Berro
Marla Berry
David Berry
Hugues Berry
Richard Bertram
Samuel Beshers
Guillaume Beslon
Alex Best
Robert Best
Sven Bestmann
Richard Betzel
Thijs Beuming
David Bevan
Andreas Beyer
Marianne Bezaire
Anand Bhaskar
Guo-Qiang Bi
Dapeng Bi
Concha Bielza
Yazan Billeh
Élise Billoir
Marc Birtwistle
William Bishop
Kyle Bittinger
Sebastian Bitzer
Martin Blackledge
Rachael Blair
Judith Blake
Rainer Blasczyk
Michael Blinov
Spencer Bliven
Sally Blower
Michael Blum
Daniel Blustein
Nils Blüthgen
Ulrich Bodenhofer
Rainer Boeckmann
Lies Boelen
Alexander Boettcher
Rafal Bogacz
Carol Boggs
Gib Bogle
Ingo Bojak
Peter Bolhuis
Erik Bollt
Hamid Bolouri
Luca Bolzoni
Peter Bond
Maciej Boni
Richard Bonneau
Tjeerd Boonstra
Victoria Booth
Luís Borda-De-Agua
Sergio Bordel
Elhanan Borenstein
Christoph Borgers
Richard Born
Aaron Bornstein
Jonathan Borrelli
Alex Borst
David Bortz
Amitabha Bose
Arezki Boudaoud
Anne-Laure Boulesteix
Peter Bourke
Alain Bousquet-Mélou
Bastien Boussau
Gregory Bowman
Scott Boyken
Ivana Bozic
Philip Bradley
Samuel Brand
Bruce Brandhorst
Jochen Braun
Michelle Brazas
Nicolas Bredeche
Felix Breden
Rainer Breitling
Wieland Brendel
Paul Bressloff
Romain Brette
Bas Breukelen
Olivier Briët
Fiona Brinkman
John-Stuart Brittain
Yana Bromberg
Jerry Bromenshenk
Bindi Brook
John Brookfield
Angela Brooks
Charles Brooks III
Mark Broom
Sam Brown
Celeste Brown
C. Titus Brown
Michael Browning
Frank Bruggeman
Nicolas Brunel
Elizabeth Brunk
Eleanor Brush

Michal Brylinski
Javier Buceta
Philipp Bucher
Nicolae-Viorel Buchete
Matthias Buck
Gavin Buckingham
Michael Buhnerkempe
Michael Buice
Catalin Buiu
Edward Bullmore
Carol Bult
Chris Burgess
Marie Burns
Dennis Burton
Zachary Burton
Tim Buschman
Giovanni Bussi
Adam Butler
Helen Byrne
Danilo Bzdok
Joana Cabral
Santiago Cadena
Hayriye Cagnan
David Cai
Peter Caie
Tunahan Cakir
Vince Calhoun
Daniela Calvetti
Anton Camacho
Brian Camley
Matthew Campbell
Stuart Campbell
Colin Campbell
Monica Campillos
Paulo Campos
Anne-Claude Camproux
Mauricio Canals
Carmen Canavier
Laetitia Canini
Mark Cannell
Yang Cao
Enrico Capobianco
Emidio Capriotti
Lucia Carbone
Peter Carbonetto
Erick Cardenas Poire
Lucas Carey
Vincent Carey
Paolo Carloni
David Carlson
Heather Carlson
Carlos Carmona-Fontaine
Laurel Carney
James Carothers
Jose Antonio Carrillo
Paola Carrillo-Bustamante
Joao Carvalho
Rita Casadio
Gastone Castellani
Carlos Castillo-Chavez
Mario Castro
Duccio Cavalieri
Bernard Cazelles
Maurice Chacron
Benny Chain
Jayprokas Chakrabarti
Saikat Chakrabarti
Jennifer Cham
Samuel Chamberlain
Hue Chan
Chia-En Chang
Chuan-Hsiung Chang
Belinda Chang
Claudine Chaouiya
Mark Chaplain
Brad Chapman
Adam Charles
Steven Chase
Krishnendu Chatterjee
P-L Chau
Rishidev Chaudhuri
Balagopalakrishna Chavali
Gal Chechik
Chia-Yen Chen
Xiaojie Chen
Ming Chen
Jianhan Chen
Xing Chen
Luonan Chen
Hua Chen
Rong Chen
Alan Cheng
Feixiong Cheng
Yuanhua Cheng
Tiejun Cheng
Jianlin Cheng
Ryan Cheng
Elizabeth Cherry
Mike Cherry
Nicholas Chia
Anush Chiappino-Pepe
Daniel Chicharro

Lauren Childs
Rebecca Chisholm
Hsuan-Chao Chiu
Anton Chizhov
Dmitrii Chklovskii
Leon Chlon
Dongyeon Cho
Jae Young Choi
Marc Choisy
Gaurav Chopra
Ching-Shan Chou
Carson Chow
Debashish Chowdhury
Gerardo Chowell
David Christini
Xi Chu
Ho-Ryun Chung
Moo Chung
Bong Jae Chung
Thomas Churcher
Stanca Ciupe
Jean Clairambault
Colleen Clancy
Hannah Clapham
Peter Clark
Timothy Clark
Richard Clark
Richard Clayton
Andrew Clayton
Yves Clément
Tyler Cluff
Alicia Clum
Dana Clutter
Sarah Cobey
Simona Cocco
Robert Cockrell
Luc Coffeng
Nick Cogan
Ted Cohen
Netta Cohen
Roi Cohen Kadosh
Vlad Cojocaru
Carlo Colantuoni
Michael Cole
Caroline Colijn
Vittoria Colizza
Matthew Collett
Anne Collins
Giorgio Colombo
M. Angels Colomer
Hans Colonius
Robert Colvin
Karen Conneely
Rory Conolly
Jessica Conway
Roger Cooke
Michael Cooling
Stephen Coombes
Leighton Core
Anne Cori
Philip Corlett
Manuel Corpas
James Costello
Michiel Cottaar
Antoine Coulon
Alexandre Courtiol
Evangelos Coutsias
Lindsay Cowell
Lenore Cowen
Katharine Coyte
Gheorghe Craciun
Keith Crandall
Darren Creek
John Cressman
Frédéric Crevecoeur
Max Crispin
David Croucher
James Crowe Jr.
Holk Cruse
Albert Cruz
Jozsef Csicsvari
Attila Csikász-Nagy
Yuwei Cui
Kathleen Cullen
John Cunningham
Will Cupples
Scott Currie
Carina Curto
Tomer Czaczkes
Andras Czirok
Lilia Da Costa
Yuri Dabaghian
Kam Dahlquist
Alma Dal Co
Chiara Dalla Man
Benjamin Dalziel
Egidio D'Angelo
Bryan Daniels
Gaudenz Danuser
Phuong Dao
Arvin Dar
Aaron Darling

Chantal Darquenne
Jayajit Das
Sayoni Das
Tommaso D'Aste
Xavier Daura
Miles Davenport
Stephen David
Lawrence David
Lance Davidson
Allan Davis
Nathaniel Daw
Adriana Dawes
Troy Day
Judy Day
Allen Day
Peter Dayan
Subhajyoti De
Daniela De Angelis
Walter de Back
Tjaart de Beer
Vincent de Gardelle
Chris de Graaf
Xavier de la Cruz
Paolo De Los Rios
Angels De Luis Balaguer
Joao de Magalhaes
Andrea De Martino
Celso de Melo
Silvia De Monte
Dick de Ridder
Jeroen de Ridder
Aymar De Rugy
Ines de Santiago
Erik De Schutter
Charlotte Deane
Florence Debarre
Nathalie Declerck
Eric Deeds
Maxime Deforet
Victor DeGruttola
Yves Dehouck
Paolo Del Giudice
Sara Del Valle
Krisine DeLeon-Pennell
Ioannis Delis
Wim Delva
Thomas Demarse
Micah Dembo
Hanneke Den Ouden
Susan Denham
James Dennis
Nicolas Desprat
Mathieu Desroches
Alain Destexhe
Andreas Deutsch
Xin Di
Diego Di Bernardo
Antonella Di Pizio
Stefano Di Talia
Mederic Diard
Jose Diaz
Frederic Dick
Alexander Dickson
Markus Diesmann
Frank Dimaio
Christopher Dimattina
Alexander Dimitrov
Feng Ding
Peter Dittrich
Marcus Dittrich
J. Brandon Dixon
Marko Djordjevic
Takahiro Doi
Nikolay Dokholyan
Socrates Dokos
Eytan Domany
Matthieu Domenech De Cellès
Brian Dominy
Alberto D'Onofrio
I. Dorigatti
Zsuzsa Dosztányi
Pamela Douglas
Christopher Douville
Andrew Doxey
Kenji Doya
Andreas Dräger
Jeremy Draghi
John Drake
Dirk Drasdo
Patrick Drew
Frank Drews
Guillaume Drion
Ron Dror
Christopher Drovandi
Shaul Druckmann
Jan Drugowitsch
Julia Drylewicz
Renato Duarte
Jessica Dubois
Eugene Duff
Craig Duffy
Michel Dumontier

Stanislaw Dunin-Horkawicz
Mary Dunlop
Geneviève Dupont
Lionel Dupuy
Salvador Dura-Bernal
Miquel Duran-Frigola
Omer Dushek
Jonathan Dushoff
James Eason
Oliver Ebenhoeh
Hermann Eberl
Julian Echave
Alexander Ecker
Philip Eckhoff
Herbert Edelsbrunner
Leah Edelstein-Keshet
Olle Edholm
Todd Edwards
Ulrich Egert
Robert Egger
Rosalind Eggo
Stephen Eglen
Tobias Egner
Gaute Einevoll
Thomas Eiting
Anna Eklof
Meriem El Karoui
Seif Eldawlatly
James Elder
Ardith El-Kareh
Timothy Elston
Valentina Emiliani
Scott Emmons
Thierry Emonet
David Enard
Jan Engelstädter
Bernhard Englitz
Markus Engstler
Magnus Enquist
Emilia Entcheva
Radek Erban
A. Murat Eren
Wolfram Erlhagen
Yaniv Erlich
Burak Erman
Udo Ernst
Jason Ernst
Rachel Errington
Adam Ertel
Serpil Erzurum
Dario Estrin
Roger Evans
William Evans
Jan Felix Evers
Stephan Ewert
James Faeder
Luca Faes
Ashkaan Fahimipour
Martin Falcke
Hai Fang
Iman Farasat
Anne Farewell
Nuno Faria
José Faria
Farzad Farkhooi
Karoline Faust
Dmitry Fedosov
Michael Feig
Ofer Feinerman
Adam Feist
Jan Feldheim
Jacob Feldman
Anatol Feldman
David Fell
Scott Feller
Jean-Marc Fellous
James Feng
Flavio H. Fenton
Manuela Ferracin
Eliseo Ferrante
Christopher Fields
Ila Fiete
C. Alberto Figueroa
Michele Filannino
Arseny Finkelstein
James Finley
Emily Finn
Gianfranco Fiore
Brian Fischer
Mareike Fischer
Andras Fiser
David Fisman
Ferdinando Fiumara
Patrick Flaherty
Ronan Fleming
Alexander Fletcher
Jonathan Flombaum
Ana Maria Florescu
Aaron Fogelson
Christopher Fonnesbeck
Bertrand Fontaine
Lorenzo Fontolan

Jasmine Foo
Patrick Forcelli
Elia Formisano
Pau Formosa-Jordan
Alex Fornito
Guillaume Fournié
Michael Francis
Paul Francois
Michael Frank
Mathias Franz
Christophe Fraser
Franca Fraternali
B.J. Fregly
Clark Freifeld
Leon French
John Fricks
Leonid Fridlyand
Iddo Friedberg
Jonathan Friedman
Nir Friedman
Ran Friedman
Benjamin Friedrich
Pascal Fries
Karl Friston
Chris Frith
Flavio Frohlich
Holger Fröhlich
Simon Frost
John Fryxell
Feng Fu
Edvin Fuglebakk
Tomoki Fukai
Laura Fumanelli
Akira Funahashi
Isaac Chun-Hai Fung
Nicholas Furnham
Terry Gaasterland
Marcal Gabalda
Bruno Gaeta
Eamonn Gaffney
Jonas Gaiarsa
Randy Gallistel
Oxana Galzitskaya
Nikita Gamper
Paolo Gandellini
Gowrishankar Ganesh
Surya Ganguli
Vitaly Ganusov
Xin Gao
Vytautas Gapsys
Thomas Garcia
Hernan G. Garcia
Jose Manuel Garcia Aznar
Hector Garcia Martin
Andy Gardner
Marta Garrido
Katharina Gaus
Philippe Gaussier
Sergey Gavrilets
Christophe Geldmacher
Lendert Gelens
Robert Gentleman
Anthony George
George Georgiou
André Gerber
Ulrich Gerland
Philip Gerlee
Mark Gerstein
Wulfram Gerstner
Francesco Gervasio
David Gfeller
Martin Giese
Peter Gillespie
Jesse Gillis
Matthieu Gilson
Jesus Giraldo
Jean-Antoine Girault
Simon Giszter
Anthony Gitter
Erida Gjini
Enrico Glaab
Jacob Glanville
Margaret Glasner
Enrico Glerean
Greg Gloor
Florian Gnad
Alberto Gobbi
Yana Gofman
Tatyana Goldberg
Mark Goldman
Edward Goldstein
Anna Golebiewska
Tim Gollisch
Leonardo Gollo
David Golomb
Jorge Gomez Tejeda Zanudo
Gerhard Gompper
Norberto Gonçalves
Mehmet Gonen
Pulin Gong
Joaquín Goñi
Didier Gonze

Benjamin Good
Marc Goodfellow
Steven Goodreau
Raluca Gordan
Jeff Gore
Alemayehu Gorfe
Cara Gottardi
Alexandros Goulas
Pierre-Antoine Gourraud
Nir Gov
Sidhartha Goyal
Niels Grabe
Carlos Gracia-Lazaro
Alan Grafen
Lyle Graham
Bruce Graham
Francois Graner
Pascal Grange
Frauke Gräter
Michael Graupner
Julien Gravier
Frederik Graw
Jeremy Green
Benjamin Greenbaum
Casey Greene
Michael Greene
Eric Greenwald
Megan Greischar
Anna Greka
Richard Grenfell
Bastian Greshake
Jamie Griffin
Timothy Griffin
Sergei Grigoryev
Jacopo Grilli
Ramon Grima
Yrjo Grohn
Alexander Grosberg
Alan Grossfield
Christina Grozinger
Matthew Grubb
Sergei Grudinin
Sonja Gruen
Wanjun Gu
Liqun (Andrew) Gu
Yuanfang Guan
John Guckenheimer
Jeremie Guedj
Emmanuel Guigon
Caterina Guiot
Rudiyanto Gunawan
Jeremy Gunawardena
Emily Gurley
Ryan Gutenkunst
William Gutheil
Robert Gütig
Giorgio Guzzetta
Melissa Gymrek
Sandor Gyorke
Jurgen Haanstra
Jürgen Haas
Julie Haas
Christian Habeck
Jason Hackney
Lilach Hadany
Neal Haddaway
Ralf Haefner
Dieter Haemmerich
Melissa Haendel
Hiroshi Haeno
Ziad Hafed
Tzachi Hagai
Michael Hagan
Steve Hahn
Richard Hahnloser
Vincent Hakim
Matthew Hall
Olivier Hamant
Joshua Hamilton
Ming Hammond
Jim Hanan
Penelope Hancock
Andreas Handel
Erik Hanschen
Elsa Hansen
Ulrich Hansmann
Alison Harrill
Heather Harrington
Andrew Harris
Todd Harris
Samuel Harrison
Matthew Harrison
Paul Harrison
Matthew Hartman
Jan Hasenauer
Gal Haspel
Vassily Hatzimanikatis
Bernard Haubold
Jason Haugh
Stefan Häusler
Peter Haverty
Ian S. Haworth

Etay Hay
Satoru Hayasaka
David Hayman
John-Dylan Haynes
Yong He
Chuan He
Qixin He
Qianchuan He
Lenwood Heath
Steffen Heber
Laurent Hébert-Dufresne
Dieter Heermann
Matthias Heinemann
Mikko Heino
Michael Heinz
Rebecca Heise
Moritz Helias
Marc Hellerstein
Richard Henchman
Adam Hendricks
Denise Henriques
Craig Henriquez
Christopher Henry
Niel Hens
Burkhard Hense
Richard Henson
Joshua Herbeck
James Herbert-Read
Enrique Hernandez-Lemus
Esteban Hernandez-Vargas
Markus Herrgard
Uri Hershberg
John Hertz
Tomer Hertz
David Herzfeld
Kathryn Hess
Holger Heyn
Stephanie Hicks
Winston Hide
Paul Higgs
Alison Hill
Daniel Himmelstein
Michael Hinczewski
Laura Hindersin
Yoshito Hirata
Mark Histed
William Hlavacek
Oliver Hobert
Emma Hodcroft
Stefan Hoehme
Thomas Höfer
Gabriel Hoffman
Kay Hofman
Martin Hofmann-Apitius
Beth Hogans
Arun Holden
Scott Holley
David Holloway
Liisa Holm
William Holmes
Susan Holmes
Philip Holmes
Hermann-Georg Holzhütter
Gary Hon
Bo Hong
Ha Hong
Jan Hontelez
Tomas Höök
Sahand Hormoz
Fabian Horn
Tiffany Horng
Amnon Horovitz
Markus Hoth
Marc Howard
Hua Hu
Zheng Hu
Yiming Hu
Chun-Hsi Huang
Haiping Huang
Xuhui Huang
Gary Huber
John Huguenard
Mark Humphries
Thomas Hund
Eric Huseby
Samer Hussein
Matthew Hutchinson
Ed Huttlin
Wonmuk Hwang
Alexandre Hyafil
Olivier Hyrien
Jang Hyunbum
Aapo Hyvarinen
Marta Ibañes
Chris Illingworth
Risto Ilmoniemi
Robin Ince
Brandon Invergo
Christos Ioannou
Bogdan Iorga
Ivan Iossifov
Zamin Iqbal

Jaime Iranzo
Manuel Irimia
Shin Ishii
Iaroslav Ispolatov
Johnny Israeli
Robert Ivanek
Ivaylo Ivanov
Natalia Ivanova
Jun Izawa
Leighton T. Izu
Jesse Jackson
Donald Jacobs
Robert Jacobs
Vincent Jacquemet
Johannes Jaeger
Dieter Jaeger
Hartmut Jaeschke
Andrew Jaffe
Samie Jaffrey
Harsh Jain
John James
Neema Jamshidi
Kevin Janes
Sarath Chandra Janga
Julian Jara-Ettinger
William Jeck
Constance J. Jeffery
Danyel Jennen
Ole Jensen
Oliver Jensen
Paul Jensen
Antoine Jerusalem
Changge Ji
Peilin Jia
Zhenyu Jia
Yi Jiang
Anupama Jigisha
Alexandra Jilkine
Jenia Jitsev
Colin Johnson
Heath Johnson
Gregory Johnson
Matthew Johnson
Vladimir Jojic
Mario Jolicoeur
Thibaut Jombart
Peter Jonas
Claus Jørgensen
Hiren Jitendra Joshi
Krešimir Josic
Daniel Jost
Paula Jouhten
David Juan
Timothy Julian
Simon Jupp
Davor Juretic
William Jusko
Nick Juty
Peter Kabos
Mads Kærn
Yariv Kafri
Daniel Kahn
Marcus Kaiser
Olga Kalinina
Stiliyan Kalitzin
Christel Kamp
Andrey Kan
Shuli Kang
Ingmar Kanitscheider
Srinivasaraghavan Kannan
Jonathan Kao
Achillefs Kapanidis
Phillipp Kapranov
Slim Karkar
Christof Karmonik
George Karniadakis
Peter Karp
Jonathan Karr
Peter Kasson
Eleni Katifori
Mamoru Kato
Steffen Katzner
Aris Katzourakis
Kamran Kaveh
Hokto Kazama
Dukka KC
Ruian Ke
Amy Keating
Brendan Keating
Lindsay Keegan
Michael Keiser
Miklos Kellermayer
David Kelley
Ross Kelly
Janet Kelso
William Kendall
Henry Kennedy
Peter Kennelly
Eamonn Keogh
Thomas Kepler
Mehdi Keramati
Eduard Kerkhoven

Leah Keshet
Can Kesmir
Beyrem Khalfaoui
Syma Khalid
Mehdi Khamassi
Sagar Khare
Hossein Khiabanian
Kamran Khodakhah
Tommy Khoo
Ekta Khurana
Natalia Khuri
Roozbeh Kiani
Warren Kibbe
Stefan Kiebel
Timothy Killingback
James Kilner
Alastair Kilpatrick
Zachary Kilpatrick
Philip Kim
Taeyoon Kim
Joonhoon Kim
Seyoung Kim
Minsu Kim
Kyoung Kim
Eunjung Kim
Michael King
Zachary Andrew King
Ross King
Carl Kingsford
Justin Kinney
David Kirchman
Denise Kirschner
Istvan Kiss
Gunnar Klau
Jeffery Klauda
Frederick Klauschen
Eili Klein
Dmitri Klimov
Don Klinkenberg
Edda Klipp
Edda Kloppmann
Johanna Klughammer
Sebastian Kmiecik
Melissa Knothe Tate
David Knowles
Andrew Knox
Beatrice Knudsen
Juergen Koefinger
Peter Kok
Georgios Kokkoris
John Koland
Peter Kolb
Artemy Kolchinsky
Maarten Kole
Andrzej Kolinski
Nils Kolling
Sepp Kollmorgen
Anatoly Kolomeisky
Michal Komorowski
Alexandr Kornev
Alon Korngreen
Andrew Kossenkov
Rumen Kostadinov
Constantinos Koumenis
Roger Kouyos
Michael Koval
Axel Kowald
Mehmet Koyuturk
Dima Kozakov
Moritz Kraemer
Danica Kragic
Mark Kramer
Peter Kramer
Matthias Krause
Anna Kreshuk
Mirjam Kretzschmar
Thomas Kreuz
Skirmantas Kriaucionis
Sandeep Krishna
Adarsh Krishnamurthy
Prashant Krishnamurthy
J. Krishnan
Anders Krogh
Joachim Krug
Martin Krupa
Andrzej Kudlicki
Lars Kuepfer
Thomas Kuhlman
Rahul Kulkarni
Supriya Kumar
Arvind Kumar
Niraj Kumar
Ádám Kun
Anshul Kundaje
James Kunert
Arthur Kuo
Yasutaka Kurata
Gen Kurosawa
Zeb Kurth-Nelson
Edo Kussell
Zoltán Kutalik
Jason Kutch

Nobuyuki Kutsukake
Kwame Kutten
Alexey Kuznetsov
Wing Hing Paul Kwan
Alain Laederach
Kevin Lafferty
Spencer Lake
Avantika Lal
Guillaume Lambert
Renaud Lambiotte
Christian Landry
David Landsman
Michael Landy
Benjamin Langmead
Filip Lankas
Anders Lansner
Jean-François Lapierre
Markus Lappe
Stephen Larson
Erik Larsson
Keren Lasker
Mario Latendresse
Ken Lau
Paul Laurienti
Kenneth Laurita
Richard Lavery
Jack Lawler
Conor Lawless
Michael Lawrence
Fides Lay
Matthew Lazzara
Mikhail Lebedev
Urszula Ledzewicz
Su-In Lee
Robin Lee
Sangwan Lee
David Lee
Ha Youn Lee
Insuk Lee
Philippe Lefèvre
Marc Lefranc
Laurent Lehmann
Ben Lehner
Ming Lei
Christian Leibig
Joel Leibo
Chris-Andre Leimeister
Jeremy Leipzig
Mark Leiserson
Christopher Lemmon
Pierre-Pascal Lenck-Santini
Mate Lengyel
Boris Lenhard
Michael Lerner
Andres Lescano
Erez Levanon
Gabriel Leventhal
Scott Levin
Anna Levina
Joel Levine
Erel Levine
Herbert Levine
Yaakov Levy
Ifat Levy
Richard Lewis
Nathan Lewis
Jarrod Lewis-Peacock
Joel Lexchin
Yue Li
Hu Li
Wenyuan Li
Zheng Li
Shuyan Li
Yichao Li
Pan Li
Jingjing Li
Jie Liang
Po-Huang Liang
Hualou Liang
Shuohao Liao
Eric Libby
Benedicte Lie
Wolfram Liebermeister
Ted Liefeld
Gabriele Lillacci
Timothy Lillicrap
Sol Lim
Sukbin Lim
Yu-Shan Lin
Chin-Hsuan Lin
Xiaoxia Lin
Kevin Lin
Scott Linderman
Rune Linding
Benjamin Lindner
Tom Lindström
Miia Lindström
Michal Linial
Andreas Linninger
Sébastien Lion
Jan Lipfert
Christoph Lippert

Jacob Litman
Weidong Liu
Tao Liu
Xiaole Shirley Liu
Yi Liu
Zhirong Liu
Yang-Yu Liu
Tianyun Liu
Jin Liu
Dennis Livesay
Adi Livnat
Joseph Lizier
Salvador Lladó
Manuel Llinas
Kevin Lloyd
Alexander Lobkovsky
Ken Locey
Eric Lock
James Locke
Gerald Loeb
Po-Ru Loh
Eric Londin
Mickey London
Nir London
Manyuan Long
André Longtin
Simone Lopes-Herrera
Carlos Lopez
Tommaso Lorenzi
Sylvie Lorthois
Ard Louis
Yoram Louzoun
Claude Loverdo
Songjian Lu
Ting Lu
Zhiyong Lu
Shaoyong Lu
Shaoying Lu
Long Lu
Vassiliy Lubchenko
Fabio Luciani
Artur Luczak
Steven Ludtke
E. Luebeck
Aaron Lun
Claudia Lunghi
Zewei Luo
Ji Luo
Christian Lüscher
George Lykotrafitis
Grant Lythe
Katrina Lythgoe
Jianzhu Ma
Jun Ma
Liya Ma
Wenzhe Ma
Shuangge Ma
Eric Ma
Jian Ma
Wei Ji Ma
Shisong Ma
Buyong Ma
Jeffrey Macdonald
Boris Macek
Christian Machens
Geoff Macintyre
Steven Mack
Paul Macklin
Adam Maclean
Sundararajan V. Madihally
Kazuhiro Maeshima
Donna Maglott
Cara Magnabosco
Carsten Magnus
Gesham Magombedze
Radhakrishnan Mahadevan
Christopher Maher
Shaun Mahony
Manuel Mai
Tiago Maia
Thomas Mailund
Francois Major
Jean-François Mangin
Michael Manhart
Richard Mann
Keefe Manning
Jeremy Manning
Michael Manolidis
Sheref Mansy
Massimo Mantegazza
Manu Manu
Sayed-Amir Marashi
Daniel Marbach
Marina Marcet-Houben
Anna Marciniak-Czochra
Daniel Marcu
Eve Marder
Adil Mardinoglu
Davide Marenduzzo
Felix Margadant
Antoni Margalida
Robert Marino

Eric Maris
Carsten Marr
Gary Marsat
Joseph Marsh
Pier Luigi Martelli
Jennifer Martin
Guillaume Martin
Andrew Martin
Pamela Martinez
Luis Martinez
Alfonso Martinez-Arias
Francisco Martínez-Jiménez
Encarnacion Martinez-Salas
Araks Martirosyan
Andriy Marusyk
Christopher Marx
Daniel Masison
Jeremy Mason
Timothée Masquelier
Paolo Massobrio
Naoki Masuda
Anthony Mathelier
Alison Mather
Nicholas Mathis
Alexander Mathis
Christoph Mathys
Laura Matrajt
Satoshi Matsuoka
Marcelo Mattar
Piel Matthieu
Maurizio Mattia
Victor Matveev
Richard Maude
Marco Mauri
Gerrit Maus
Philip Maybank
J. Patrick Mayo
Borbala Mazzag
Alexander Mcavoy
Kim McCabe
Kevin McCairn
David McCandlish
Andrew McDavid
Josh McDermott
Jason McDermott
Thomas McDonald
Mark McDonnell
Johanna McEntyre
Hassane Mchaourab
Richard McNally
Daniel McNamee
Shannon McWeeney
Marmar Mehrabadi
Mehrdad Mehrbod
Sohum Mehta
Ron Meir
Andrew Melbourne
Francisco Melo
Raoul-Martin Memmesheimer
Pedro Mendes
Josh Merel
Roeland Merks
Stefano Merler
Frederic Mery
Nicolette Meshkat
Jill Mesirov
Christian Metallo
Dirk Metzler
Antonia Mey
Arne Meyer
Diogo Meyer
Martin Meyer
Michael Meyer-Hermann
Pieter Meysman
Mihaly Mezei
Qi Mi
Hongyu Miao
George Michailidis
Melchi Michel
Andrea Micheletti
Nicole Mideo
Tam Mignot
Stefan Mihalas
Jason Mikiel-Hunter
Shawn Mikula
Lorin Milescu
Andreas Milias-Argeitis
Joel Miller
Martin Miller
Paul Miller
Kyle Miller
Gary Mirams
Leonid Mirny
Konstantin Mischaikow
Bratislav Misic
Katie Mitchell-Koch
Jeetain Mittal
Wayne Mitzner
Takashi Miura
Yin-Yuan Mo
David Mobley
Mohammad Mofrad

Alex Mogilner
Sonali Mohanty
Oliver Mohr
Mark Moll
Gianluigi Mongillo
Stephen Montgomery
Luca Monticelli
Sean Mooney
James Moore
Brian Moore
Thierry Mora
Mahmoud Moradi
Juan Morales
Rosalyn Moran
Ion Moraru
Ryan Morehead
Yamir Moreno
Ruben Moreno-Bote
Miguel Moreno-Risueno
Martin Morgan
Susumu Mori
Stefano Morotti
Giulia Morra
Edward Morrissey
Mathieu Moslonka-Lefebvre
Thiago Mosqueiro
Robert Moss
Sara Mostafavi
Allan Motyer
Alessandro Moura
Zissimos Mourelatos
Ahmed Moustafa
Shang Mu
Andrew Mugler
Arnab Mukherjee
Shayantani Mukherjee
Sayan Mukherjee
Sarah Muldoon
Viktor Müller
Timothy Mullett
Christopher Mungall
Lance Munn
Jose Munoz
Marieke Mur
John Murray
Philip Murray
Samuel Muscinelli
Ferdinando Mussa-Ivaldi
Michael Muthukrishna
Christopher Myers
Nicholas Myers
Chris Myers
Suhita Nadkarni
Swati Nagar
John Nagy
Armaghan Naik
Honda Naoki
Lucia Napione
Rishikesh Narayanan
Jatin Narula
Jeremie Naude
Saket Navlakha
Zaneta Navratilova
Sriram Neelamegham
Keith Neeves
Richard Neher
Paul Nelson
Martin Nelson
Ilya Nemenman
Bernhard Nessler
Theoden Netoff
Heiko Neumann
Susana Neves
Jonathan Newton
Michael Newton
David Ng
Pauline Ng
Dao Nguyen
Benjamin Nichols
Steven Niederer
Elisabet Nielsen
Morten Nielsen
Noushin Niknafs
Viacheslav Nikolaev
Zoran Nikoloski
Vadim Nikulin
Lennart Nilsson
Mahesan Niranjan
Hiroshi Nishiura
Aleksandra Nita-Lazar
Clement Nizak
Robert Noble
Matthew Nolan
Taishin Nomura
Elad Noor
Yohei Norimatsu
Sergei Noskov
Richard Notebaart
Armita Nourmohammad
Houtan Noushmehr
Pierre Nouvellet
Thomas Nowotny

Daichi Nozaki
Johannes Nuebler
Ana Nunes
Jessica Oakes
Patrick Oakes
Terrence Oas
Uri Obolski
Sean O'Callahan
Alejandro Ochoa
Mike Ochs
Gabriel Ocker
Brian Odegaard
Cian O'Donnell
James O'Dwyer
Dietmar Oelz
Thomas O'Hara
Uwe Ohler
Michael Oldham
Andres Olivares
Guilherme Oliveira
Bruno Olshausen
Mette Olufsen
Paul Omaille
Gilbert Omenn
Nooshin Omranian
Mary Jo Ondrechen
Lulla Opatowski
Lance Optican
Jean-Jacques Orban De Xivry
Yaron Orenstein
Enzo Orlandini
Remus Osan
Roman Osman
Alexei Ossadtchi
Andrew Oster
Andrei Osterman
Arne Ostman
Srdjan Ostojic
David Ostrov
Niels Otani
Hans Othmer
Sarah Otto
Markus Owen
Shuji Ozaki
Ertugrul Ozbudak
Banu Ozkan
Elif Ozkirimli Olmez
Alberto Paccanaro
Marius Pachitariu
Karen Page
Joris Paijmans
Gergely Palla
Stephanie Palmer
Lucy Palmer
Thomas Palmeri
Wei Pan
Orestis Panagiotou
Anna R Panchenko
Om Pandey
Sandeep Pandit
Alexander Panfilov
Liam Paninski
Casian Pantea
Stefano Panzeri
Elena Papaleo
Garegin Papoian
Kamal Raj Pardasani
Andrew Park
Il Park
John Parkinson
Julia Parrish
Srinivasan Parthasarathy
Alexander Pastukhov
Kiran Patil
Pintu Patra
Sinu Paul
Jonas Paulsen
Paul Pavlidis
Klaus Pawelzik
Samuel Payne
Joshua Payne
William Pearson
Morten Pedersen
Bradford Peercy
Shayn Peirce
Lucia Peixoto
Serge Pelet
Jorge Peña
Roger Peng
Jian Peng
Huiming Peng
Zhangli Peng
Melissa Penny
Matjaz Perc
Alan Perelson
Juan Perez
Alberto Perez
Alfonso Perez
Matthew Perisin
Vipul Periwal
Theodore Perkins
Alex Perkins

Andrea Perna
Arkady Pertsov
Fernando Peruani
Franco Pestilli
Reuben Peters
Megan Peters
Carsten Peterson
Giovanni Pezzulo
Jean-Pascal Pfister
Brian Pfleger
Herve Philippe
Roger Phillips
Steven Phillips
Andrew Phillips
Robert Phillips
Steven Piantadosi
Michael Pickles
Florian Pieper
Simone Pigolotti
Katherine Pillman
Jonathan Pillow
Flávio Pinheiro
Roger Pique-Regi
Eleftheria Pissadaki
Efstratios Pistikopoulos
Vincent Plagnol
Francisco Planes
Roy Platt
Adrian Platts
Jürgen Pleiss
Dietmar Plenz
Elizabeth Ploetz
Steven Plotkin
Martyn Plummer
Eftychios Pnevmatikakis
Michael Poidinger
Daniel Polani
Aleksandar Poleksic
Philip Polgreen
Thomas D. Pollard
Katherine Pollard
Dimitris Polychronopoulos
Sean Polyn
Régis Pomès
Silvina Ponce-Dawson
Margarita Pons-Salort
Anthony Poole
Hoifung Poon
Art Poon
Mihai Pop
Aleksander Popel
Michel Popoff
Jordan Poppenk
Eduard Porta-Pardo
Carol Post
David Post
Alexandre Pouget
Babak Pourbohloul
Robert Preissner
Steven Prescott
Nathan Price
Nicholas Price
Viola Priesemann
Jose Principe
Astrid Prinz
U. Deva Priyakumar
James Procter
Nicholas Provart
Davide Provasi
Thorsten Prustel
Jose Puglisi
Miquel Angel Pujana
Jeremy Purvis
Shu-Bing Qian
Zhaohui Qin
Vito Quaranta
Aaron Quinlan
T. Alexander Quinn
Diego R. Barneche
Itzhak Rabin
Joshua Rabinowitz
Mala Radhakrishnan
Ravi Radhakrishnan
Joachim Rädler
Anu Raghunathan
Arjun Raj
Markus Ralser
Indira Raman
Kasper Rand
Amanda Randles
Padmini Rangamani
Sridhar Ranganathan
Sylvia Ranjeva
James Rankin
Rajesh Rao
Wouter-Jan Rappel
Daniel Rasmussen
David Rasmussen
Matthew Rasmussen
Pasi Rastas
Thomas Rattei
Christoph Ratzke

Andreas Raue
Sarah Rauscher
Erzsébet Ravasz Regan
Christian Ray
Debashree Ray
Soumya Raychaudhuri
Jonathan Read
Mario Recker
Leila Reddy
Gregory Reeves
Florian Rehfeldt
Nicholas Reich
Udo Reichl
Juri Reimand
Michael Reimann
Matthew Reimherr
Robert Reiner
Robert Remez
Jian Ren
Alfonso Renart
Elisabeth Rens
Yakir Reshef
Olivier Restif
Boris Reva
Alex Reyes
John Reynolds
Mina Rho
Benjamin Ribba
Noah Ribeck
Ruy Ribeiro
John Rice
Daniel Rice
William Richardson
Magnus Richardson
Jonas Richiardi
Tim Ricken
José Luis Riechmann
Ingmar Riedel-Kruse
Daniel Rigden
Mattia Rigotti
Lionel Rigoux
Steven Riley
John Rinzel
Herre Jelger Risselada
Davide Risso
Marylyn Ritchie
Christian Ritz
Manuel Rivas
Elena Rivas
Denis Rivière
Olivier Rivoire
Gordon Roberston
Mark Robinson
Heath Robinson
Peter Robinson
Hugh Robinson
Gene Robinson
Walter Rocchia
Isabel Rocha
Mathieu Roche
Benjamin Roche
David Rocke
Isabel Rodriguez-Barraquer
Ignacio Rodriguez-Brenes
Raul Rodriguez-Esteban
Adrienne Roeder
Tomasz Rog
Tim Rogers
Gustavo Rohde
Ottar Rolfsson
Sandro Romani
Natalie Romanov
Rafael Romero-Garcia
Ethan Romero-Severson
Libin Rong
Peter Rose
Rebecca Rose
Gail Rosen
Rebeca Rosengaus
Edith Ross
Christian Rössert
Arnd Roth
Christian Roth
Stefan Rotter
Richard Röttger
Yasser Roudi
Joan Roughgarden
Richard Roush
Juho Rousu
Igor Rouzine
Maga Rowicka
Alex Roxin
Deodutta Roy
Sushmita Roy
Jianhua Ruan
Leonid Rubchinsky
Daniel Rubenstein
Jonathan Rubin
Alon Rubin
Volker Rudolf
Elmar Rueckert
Sten Ruediger

Jakob Ruess
Matthew Ruffalo
Eytan Ruppin
Craig Rusin
Ian Russell
Edward Ruthazer
Ueli Rutishauser
Pedro Saa
Lao Saal
Philip Sabes
Sadra Sadeh
Kashif Sadiq
Farrah Sadre-Marandi
Claude Saegerman
Julio Saez-Rodriguez
Murat Saglam
Marie-France Sagot
Rajib Saha
Erik Sahai
Maneesh Sahani
Gerald Saidel
Henrik Salje
Daniel Salomon
Nathan Salomonis
Xavier Salvatella
Michael Sammeth
Aravinthan Samuel
Nikolay Samusik
Lucas Sánchez
Thomas Sandmann
Susanna-Assunta Sansone
Fidel Santamaria
Janine Santos
Francisco Santos
Mauro Santos
Hessam Sarjoughian
Ugis Sarkans
Ram Rup Sarkar
Casim Sarkar
Venkata Satagopam
Rahul Satija
Daisuke Sato
Herbert Sauro
Thomas Sauter
Richard Savage
Natasha Savage
Nick Savill
Cristina Savin
Yonatan Savir
Gregory Sawicki
Samuel Scarpino
Stefan Schaal
William Schafer
Ralf Schäfer
Lars Schäfer
Robert Scharpf
Michael Schaub
Aaron Schein
Walter Scheirer
Michael Schellenberger Costa
Nathan Schiele
Helmut Schiessel
Steven Schiff
Birgit Schilling
Matthias Schlesner
Patrick Schloss
Jonas Schluter
John Schmidt
Christoph Schmidt-Hieber
Achim Schnaufer
Ian Schneider
Reinhard Schneider
Gisbert Schneider
Elad Schneidman
Jan Schnupp
Alexander Schoenhuth
Jacqueline Scholl
Joshua Schraiber
Gideon Schreiber
Susanne Schreiber
Michael Schroeder
Christian Schröter
André Schultz
Linus Schumacher
David Schwab
Philipp Schwartenbeck
Russell Schwartz
Cornelius Schwarz
Ulrich Schweizer
Lars Ole Schwen
Etienne Schwob
Joost Schymkowitz
Marco Scianna
Annalisa Scimemi
Celine Scornavacca
Matthew Scott
Jacob Scott
Pat Scott
Timothy Secomb
Luigi Sedda
Gunnar Seemann
David Seifert

Luc Selen
Herve Seligmann
Allen Selverston
Walter Senn
Rodolphe Sepulchre
Oliver Serang
Pier Nicola Sergi
Adrian Serohijos
M. Ángeles Serrano
Giovanni Settanni
Manu Setty
Stefano Severi
Ugur Sezerman
Pedro Sfriso
Ioannis Sgouralis
Shawn Shadden
Premal Shah
Imran Shah
Vahid Shahrezaei
Maoz Shamir
Shihab Shamma
Maryam Shanechi
Fnu Shankaracharya
Daryl Shanley
Malvika Sharan
Paul Sharp
Tatyana Sharpee
Thomas Sharpton
Eric Shea-Brown
Amarda Shehu
Karthik Shekhar
Yang Shen
Shan Shen
Stephen Shennan
Arthur Sherman
Jian Shi
Darryl Shibata
Denis Shields
Masanori Shimono
Troy Shinbrot
Shigeru Shinomoto
Tomer Shlomi
Lior Shmuelof
Ilya Shmulevich
Suzanne Shoffner
Sourya Shrestha
Stanislav Shvartsman
Sachdev Sidhu
Kyriaki Sidiropoulou
Fabian Sievers
Eric Siggia
Jessica Siltberg-Liberles
Pamela Silver
Petr Simecek
Fabio Simoes De Souza
Julie Simpson
David Sims
Shailza Singh
Mona Singh
Abhyudai Singh
Albert Siryaporn
Daniel Sitar
Jesper Sjöström
Anne Skeldon
Frances Skinner
Jane Skok
Jan Skotheim
Marcin Skwark
Malcolm Slaney
Boris Slepchenko
Paul Smaldino
Joost Smid
Timo Smieszek
Thomas Smith
Matthew Smith
James Sneyd
Samuel Sober
Eric Sobie
Johannes Söding
Peter Sokol-Hessner
Erik Solbu
Dieter Söll
Samuel Solomon
Marc Sommer
Haim Sompolinsky
Jiangning Song
Mingzhou Song
Hyun-Seob Song
Zhen Song
Xiaofeng Song
Guang Song
Chi Song
Francis Song
Swapnil Sonkusare
Nikolaus Sonnenschein
Zita Soons
Andrea Sottoriva
Hédi Soula
Victor Sourjik
Doug Speed
Andreas Spiegler
Stephanie Spielman

Jiri Sponer
Tommy Sprague
Serena Spudich
Mandyam Srinivasan
Scott Stagg
Robert Stahelin
Phillip Staniczenko
Kenneth Stanley
Oliver Stegle
Amelie Stein
Lincoln Stein
Julia Steinberg
Steven Steinway
Martin Stemmler
Greg Stephens
Michael Stern
Dagmar Sternad
Ralf Steuer
Ian Stevenson
Philip Stewart
Alexander Stewart
Jonathan Stieglitz
Brandilyn Stigler
Edward Stites
Alan Stocker
Roman Stocker
Maureen Stolzer
Chris Stone
Katherine Storrs
Roland Stote
Sebastiano Stramaglia
Daniel Streicker
Giovanni Strona
Claudio Struchiner
David Strutt
Wanda Strychalski
Michael Stubbington
Michael Stumpf
Mark Styczynski
Srikrishna Subramanian
Yukiko Sugi
Béla Suki
Pavel Sumazin
Christopher Summerfield
Mark Sun
Jingchun Sun
Edward Susko
Shinsuke Suzuki
Melody Swartz
Gyorgy Szabo
Gábor Szederkényi
Attilia Szolnoki
Henrik Szurmant
Enzo Tagliazucchi
Shoji Takada
Nobuto Takeuchi
Vicente Talanquer
Alena Talkachova
Hua Tan
Chao Tang
Yun Tang
Zhengzheng Tang
Min Tang
Yinjie Tang
Eric Tannier
Dean Tantillo
Peng Tao
Sonia Tarazona
Adi Tarca
Ann Tate
Nicholas Tatonetti
Iman Tavassoly
Merryn Tawhai
Jiannis Taxidis
James Taylor
Peter Taylor
Mark S. Taylor
Jordan Taylor
Vladimir Teif
Amalio Telenti
Pieter Rein Ten Wolde
Andrew Teschendorff
Christian Tetzlaff
Bas Teusink
Shivendra Tewari
Sharma Thankachan
Amantha Thathiah
Fabian Theis
Darryl Thelen
Raghuram Thiagarajan
Bertrand Thirion
Paul Thomas
Duncan Thomas
Andrew Thomas
Peter Thomas
Barry Thompson
Matt Thomson
Jeffrey Thorne
Kevin Thornton
Rüdiger Thul
Hans-Hermann Thulke
Bin Tian

Tianhai Tian
Xiao-Jun Tian
Chabane Tibiche
Peter Tieleman
Paolo Tieri
Paul Tiesinga
Mikhail Tikhonov
Michael Tildesley
Yulia Timofeeva
Michele Tizzoni
Gašper Tkacik
Dror Tobi
Weida Tong
Giulio Tononi
Sina Tootoonian
Joaquin Torres
Gelsy Torres-Oviedo
Silvio Tosatto
Zach Tosi
Jonathan Touboul
Hazem Toutounji
Taro Toyoizumi
Annelise Tran
Leon-Charles Tranchevent
Rui Travasso
Michael Travisano
Pauline Traynard
Beatriz Trenor
Alessandro Treves
Victor Trevino
Marcos Trevisan
Susannah Tringe
Carl Troein
Brett Trost
Wilson Truccolo
Ala Trusina
Meng-Li Tsai
Boo Shan Tseng
Konstantinos Tsetsos
Aviad Tsherniak
Lev Tsimring
Nikolaos M. Tsoukias
Naotsugu Tsuchiya
Fuchiang (Rich) Tsui
Lisa Tucker-Kellogg
Tamir Tuller
Nurcan Tuncbag
Paul Turner
Jerold Turner
John Tyson
Balazs Ujfalussy
David Umulis
Vladimir Uversky
Federico Vaggi
Nagarajan Vaidehi
Naveen Vaidya
Eugenio Valdano
Glyn Vale
Alfonso Valencia
Angelo Valleriani
Giorgio Vallortigara
Minus van Baalen
Robert van Beers
Jeremy Van Cleve
Hugo Van den Berg
Ronald van den Berg
Albert van den Berg
Wouter van den Bos
Martijn van den Heuvel
Arjan van der Vaart
John van Noort
Arjen van Ooyen
Willem Van Panhuis
Jaap van Pelt
Natal van Riel
Mark van Rossum
Carl van Vreeswijk
Gerard van Westen
Fabio Vandin
Jeffrey Varner
Gael Varoquaux
Vítor Vasconcelos
Eleni Vasilaki
Dimitrios Vavylonis
Sameer Velankar
Jorge Velasco-Hernandez
Marko Vendelin
Valerie Ventura
Ophelia Venturelli
Venessa Venturi
Robert Verity
Sergio Verjovski-Almeida
Gennady Verkhivker
Chandra Verma
L. Vermeulen
Paul Verschure
Karin Verspoor
Timothy Verstynen
Cecile Viboud
Peter Vickerman
Jean-Marc Victor
Diego Vidaurre

Mathukumalli Vidyasagar
Edward Vigmond
Mauno Vihinen
Jose Vilar
Martin Vinck
Yannick Viossat
Yoram Vodovotz
Vincent Voelz
Tim Vogels
Margaritis Voliotis
Niels Volkmann
Vitaly Volpert
Erik Volz
Erik Von Elm
Viktor Von Wyl
Dirk Vorberg
Igor Vorobyov
Gregory Voth
Gert Vriend
Wytse Wadman
Günter Wagner
Jon Wakefield
Sam Walcott
Aleksandra Walczak
Levi Waldron
Edgar Walker
Jamie Walker
Rachel Walker
Jacco Wallinga
Matthew M Walsh
Edgar Walters
Dirk Walther
Vicky Wang
Chen Wang
Wenyi Wang
Zhihui Wang
Zichen Wang
Rachel Wang
Yonghua Wang
Xiaowei Wang
Chun-Chao Wang
Zhong Wang
Wei Wang
Yingxue Wang
Xiaowo Wang
Yijie Wang
Xiao Wang
Yi Wang
Yida Wang
Lucas Wardil
Aryeh Warmflash
Silvio Waschina
Karen Watanabe
Takamitsu Watanabe
Alice Wattam
Kunlin Wei
Wenhua Wei
Martin Weigt
Cornelis Weijer
Seth Weinberg
Harel Weinstein
Alan Weinstein
Daniel Weissman
Brian Weitzner
Joshua Welch
Lonnie Welch
David Welch
Lena Welsh
Keith Weninger
Jonathan Wenk
Thomas Wennekers
Wolfgang Wenzel
Amy Weslowski
Lodewyk Wessels
Michael Wester
Nico Westerhof
Oscar Westesson
Sarah Wheelan
Nicole Wheeler
Stephen White
Michael White
Laura White
David Whitney
Steven Wiederman
Paul Wiggins
Ellen Wijsman
Bartek Wilczynski
David Wild
Avani Wildani
Brian Wilhelm
Amy Williams
Cranos Williams
Ross Williamson
Christian Wilms
Hugh Wilson
William Wilson
Klaus Wimmer
Martin Winkler
Christof Winter
Tarynn Witten
Thomas Witzel
Michal Wojciechowski

Yuri Wolf
Charles Wolgemuth
Bryan Wong
Jason Wong
Chung Wong
Kongfatt Wong-Lin
Jerome Wong-Ng
Sukyung Woo
Colin Worby
Willy Wriggers
Yinghao Wu
Yufeng Wu
Gabriel Wu
Qian Wu
Zheng Wu
Di Wu
C.-ting (Ting) Wu
Yilin Wu
Thomas Wu
Song Wu
Si Wu
Andreas Wutz
Richard Wyatt
Chris Wymant
Ruibin Xi
Jinxiang Xi
Lili Xi
Fangfang Xia
Yu Xia
Feifei Xiao
Zhengwei Xie
L. Xie
Jianhua Xing
Heng Xu
Yu Xue
Gur Yaari
Ayako Yachie-Kinoshita
Yuedong Yang
Laurence Yang
Wan Yang
Yang Yang
Guang Yao
Yosi Yarom
Andrew Yates
Yuzhen Ye
Esti Yeger-Lotem
Gene Yeo
Soojin Yi
Tau-Mu Yi
Ahmet Yildiz
Izzet Yildiz
Atsushi Yokoi
Shibu Yooseph
Lingchong You
Samuel Young
Eric Yttri
Jiyang Yu
Alan Yu
Haiyuan Yu
Jin Yu
Guoxian Yu
Guo-Cheng Yuan
Yinyin Yuan
Feng Yue
Joshua Yukich
Vasily Zaburdaev
Martin Zacharias
Osvaldo Zagordi
Bojan Zagrovic
Naif Zaman
Gorka Zamora-López
Mattia Zampieri
Krista Zanetti
Ronen Zangi
Kathi Zarnack
Veronika Zarnitsyna
Melissa Zavaglia
Christoph Zechner
Elazar Zelzer
Michael Zeng
Helen Zgurskaya
Shihua Zhang
Ning Zhang
Qingpeng Zhang
Zhiwu Zhang
Yingkai Zhang
Bin Zhang
Louxin Zhang
Jing Zhang
Zhaolei Zhang
Dong Zhang
Wei Zhang
Ni Zhao
Qi Zhao
Zhongming Zhao
Yongan Zhao
Li Zhaoping
Xiaobin Zheng
Deyou Zheng
Wenjun Zheng
Jie Zheng
Weifan Zheng

Yi Zhong
Cuncong Zhong
Shan Zhong
Xiaobo Zhou
Jian Zhou
Tong Zhou
Shanfeng Zhu
Chengsheng Zhu
Qiang Zhu
Jie Zhu
Anton Zilman
Michael Zimmermann
Johannes Zimmermann
Giuseppe Zito
Daniel Zitterbart
Noam Ziv
Michal Zochowski
Ali Zomorrodi
Jinfeng Zou
Anze Zupanic
